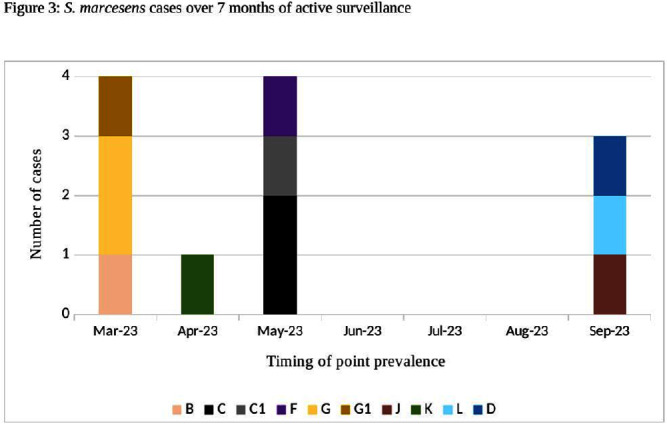# Serratia marcescens Burden in a Neonatal Intensive Care Unit: Colonization Rate, Clinical Infections and Strain Relatedness

**DOI:** 10.1017/ash.2024.333

**Published:** 2024-09-16

**Authors:** Halima Dabaja Younis, Angelie Seguban, Andrea Morillo, Tony Mazzulli, Ming Lum, Jennie Johnstone

**Affiliations:** Sinai Health System- Toronto; Sinai Health; Mount Sinai Hospital

## Abstract

**Background:** Serratia marcescens (S. marcescens) is an environmentally associated organism known for causing healthcare associated infections and outbreaks in neonatal intensive care units (NICUs). The colonization or infection rates in NICU settings remain uncertain. This study aims to evaluate the rate of baseline colonization and clinical infection and relatedness of S. marcescens isolates. **Methods:** Prospective surveillance of rectal colonization and clinical infection of S. marcescens was conducted on patients admitted to the NICU at Mount Sinai Hospital in Toronto, Ontario, from March 1, 2023, to September 30, 2023. The NICU is a 57 bed unit with all private rooms. Monthly point prevalence assessments by rectal screening were performed, alongside active surveillance for clinical infections associated with S. marcesens. Isolates from screening or clinical samples underwent assessment for relatedness using pulse field gel electrophoresis (PFGE). **Results:** Over the 7 month study period, 12 different patients (5.4%) were colonized/infected with S. marcescens. Among these, 10 patients (4.5%) were identified through rectal screening (316 rectal swabs were collected from 224 patients) and two patients (0.9%) exhibited positive clinical specimens (urine and endotracheal aspirate) in association with pyelonephritis and ventilator-associated pneumonia, respectively. Of the two clinical cases, one case showed a negative preceding rectal swab and the other detected through a clinical sample before the point prevalence date. The age at which a positive S. marcescens swab or positive clinical specimen was identified ranged from 4 to 66 days (median=18 days, IQR 5-38.8) (Figure 1). Sixty seven infants had repeated screening. Three out of 67 (4.5%) were colonized with S. marcescens, the timing and sequence of positive and negative testing are presented in Figure 2. Females demonstrated a higher positivity rate compared to males [9.1% (9/99) vs 2.4% (3/125), p=0.04, respectively]. PFGE analysis of all 12 (100%) isolates revealed a polyclonal pattern. Most cases were detected from March to May in 9/12 cases (75%). Ten different strains were identified. Notably, two strains demonstrated clusters of two cases each, one during March and the other during May (Figure 3). No mortality was reported among the cases. **Conclusions:** The study highlights the polyclonal nature of S. marcesens and raises questions about the utility of point prevalence in anticipating clinical cases or patient-to-patient transmission, especially in patients with clinical infection where there were no preceding positive screening tests.

**Figure:**
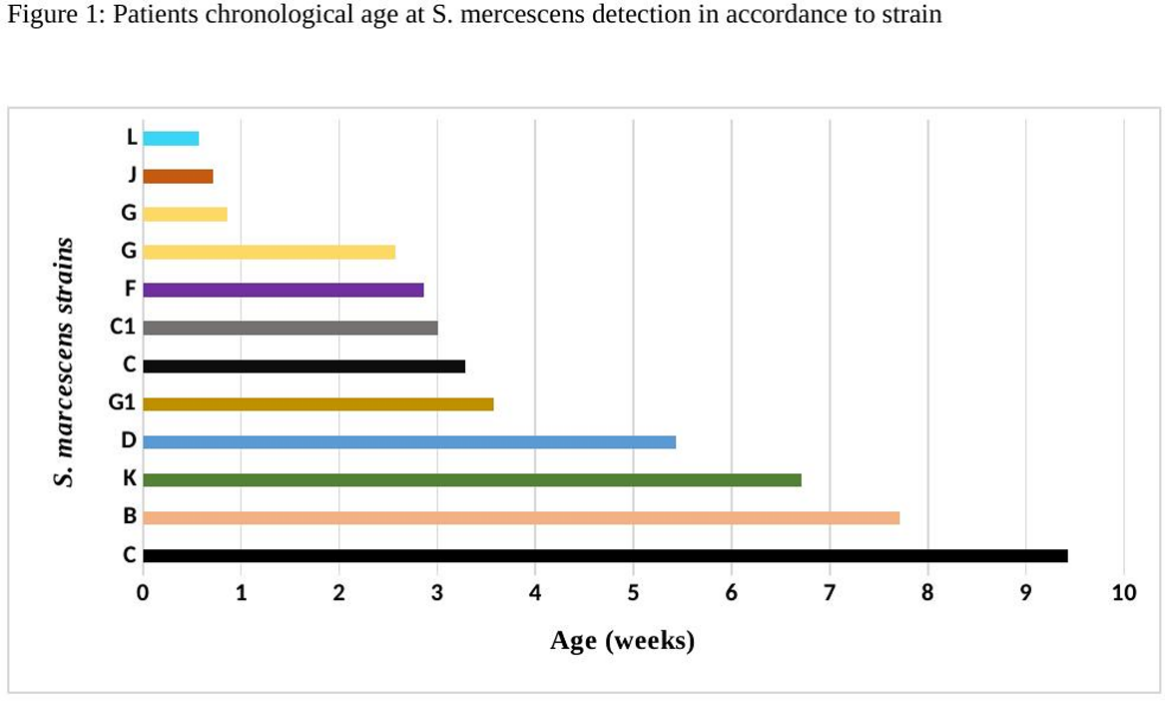

**Figure:**
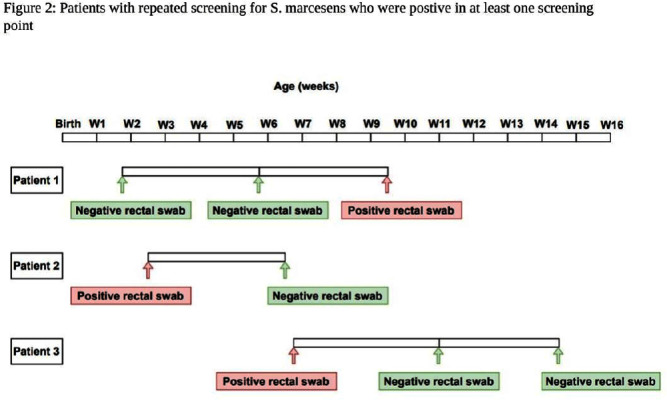

**Figure:**